# Supplementary material for: Wavelet-Based Topological Loss for Low-Light Image Denoising
Source: Sensors (Basel). 2025 Mar 25;25(7):2047. doi: 10.3390/s25072047 (PMC11990961; doi:10.3390/s25072047)
Supplement: Supplementary file 1 [file sensors-25-02047-s001.zip › sensors-3451418-supplementary.pdf]

Supplementary Materials: Wavelet-based Topological Loss for Low-Light Image Denoising

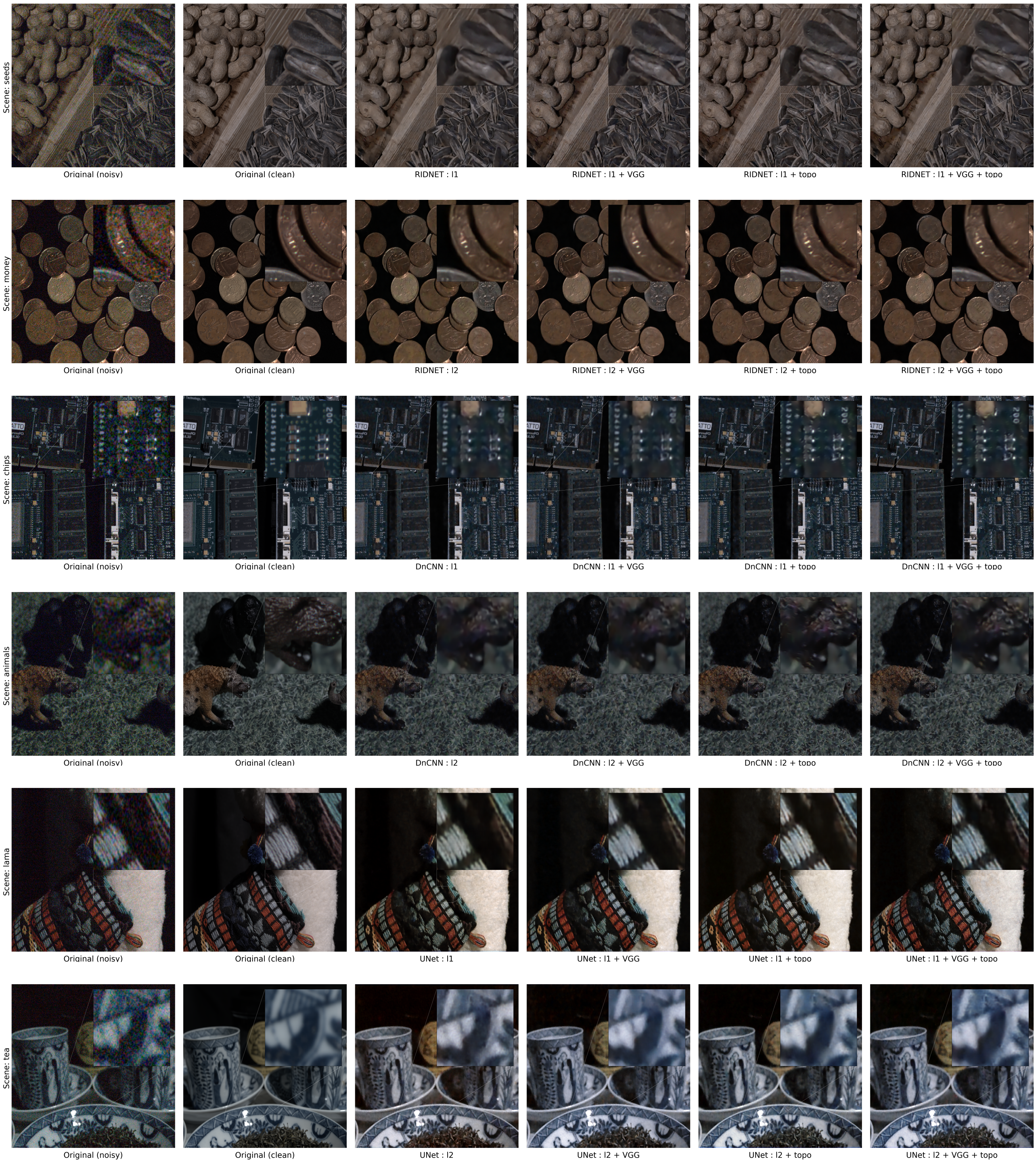

Figure S1. Subjective results of RIDNET (row 1 and 2), DnCNN (row 3 and 4) and UNet (row 5 and 6), tested on the scenes from BVI-Lowlight denoising dataset. Zoom in for better resolution.
